# Baseline structural MRI and plasma biomarkers predict longitudinal structural atrophy and cognitive decline in early Alzheimer’s disease

**DOI:** 10.1186/s13195-023-01210-z

**Published:** 2023-04-11

**Authors:** Long Xie, Sandhitsu R. Das, Laura E. M. Wisse, Ranjit Ittyerah, Robin de Flores, Leslie M. Shaw, Paul A. Yushkevich, David A. Wolk, for the Alzheimer’s Disease Neuroimaging Initiative

**Affiliations:** 1 Penn Image Computing and Science Laboratory (PICSL), Department of Radiology, University of Pennsylvania, 3700 Hamilton Walk, Suite D600, Richards Building 6th floor, Philadelphia, PA 19104 USA; 2 Penn Memory Center, University of Pennsylvania, Philadelphia, PA USA; 3 Department of Diagnostic Radiology, Lund University, Lund, Sweden; 4 Department of Pathology and Laboratory Medicine, University of Pennsylvania, Philadelphia, PA USA

**Keywords:** Biomarkers, MRI, Plasma, Tau

## Abstract

**Background:**

Crucial to the success of clinical trials targeting early Alzheimer’s disease (AD) is recruiting participants who are more likely to progress over the course of the trials. We hypothesize that a combination of plasma and structural MRI biomarkers, which are less costly and non-invasive, is predictive of longitudinal progression measured by atrophy and cognitive decline in early AD, providing a practical alternative to PET or cerebrospinal fluid biomarkers.

**Methods:**

Longitudinal T1-weighted MRI, cognitive (memory-related test scores and clinical dementia rating scale), and plasma measurements of 245 cognitively normal (CN) and 361 mild cognitive impairment (MCI) patients from ADNI were included. Subjects were further divided into β-amyloid positive/negative (Aβ+/Aβ−)] subgroups. Baseline plasma (p-tau_181_ and neurofilament light chain) and MRI-based structural medial temporal lobe subregional measurements and their association with longitudinal measures of atrophy and cognitive decline were tested using stepwise linear mixed effect modeling in CN and MCI, as well as separately in the Aβ+/Aβ− subgroups. Receiver operating characteristic (ROC) analyses were performed to investigate the discriminative power of each model in separating fast and slow progressors (first and last terciles) of each longitudinal measurement.

**Results:**

A total of 245 CN (35.0% Aβ+) and 361 MCI (53.2% Aβ+) participants were included. In the CN and MCI groups, both baseline plasma and structural MRI biomarkers were included in most models. These relationships were maintained when limited to the Aβ+ and Aβ− subgroups, including Aβ− CN (normal aging). ROC analyses demonstrated reliable discriminative power in identifying fast from slow progressors in MCI [area under the curve (AUC): 0.78–0.93] and more modestly in CN (0.65–0.73).

**Conclusions:**

The present data support the notion that plasma and MRI biomarkers, which are relatively easy to obtain, provide a prediction for the rate of future cognitive and neurodegenerative progression that may be particularly useful in clinical trial stratification and prognosis. Additionally, the effect in Aβ− CN indicates the potential use of these biomarkers in predicting a normal age-related decline.

**Supplementary Information:**

The online version contains supplementary material available at 10.1186/s13195-023-01210-z.

## Background

Curing Alzheimer’s disease (AD) and related dementias (ADRD) is one of the great challenges of our generation. It is generally accepted that interventions are likely to be most effective early in the disease course when symptoms are minimal, if present at all [1]. Therefore, the early phases of AD, i.e., preclinical or prodromal stages, are of particular interest to AD pharmaceutical research and clinical trials. Disrupting AD progression during this phase is likely to offer greater benefit to patients than interventions in later phases when extensive neurodegeneration (neuron and synapse loss) has already taken place with accompanying severe cognitive symptoms. One crucial aspect of the success of clinical trials that target early AD is to recruit participants that are more likely to progress over the course of the trials to maximize the chance of detecting a treatment effect. Hence, developing highly sensitive and specific baseline biomarkers for early AD that are predictive of longitudinal disease progression in a relatively short follow-up time frame (i.e., 5 years) will have important utility in the screening of clinical trials and participant recruitment in research studies.

Positron emission tomography (PET)- and cerebrospinal fluid (CSF)-based biomarkers of AD-related molecular pathology and neurodegeneration have proven to be useful in predicting a decline in preclinical and prodromal phases of AD [2–12]. However, blood-based biomarkers of β-amyloid (Aβ) [13, 14], p-tau [15–17], and neurodegeneration [18, 19] and structural MRI measurements [20, 21] may be more feasible in clinical trials and, eventually, practice due to convenience and cost. Prior research has demonstrated the prognostic value of these two kinds of biomarkers independently. Using structural MRI, a large number of studies have successfully predicted mild cognitive impairment (MCI) to dementia progression [22–24] and in predicting cognitive decline in MCI and/or AD dementia [2, 4, 24, 25], which have been comprehensively summarized in Weiner et al. [26]. Using MRI in cognitively normal individuals, investigators have shown that detailed measures of the medial temporal lobe (MTL) [27–31] and prefrontal cortex [32] were predictive of the development of MCI or the presence of amyloid pathology measured by CSF β-amyloid markers [33–35]. In studies focused on blood-based biomarkers, Cullen et al. demonstrated the combination of various plasma measurements, including Aβ42/40, p-tau_217_, and neurofilament light (NfL) chain, was predictive of cognitive decline and subsequent development of AD dementia (follow-up time: 4.75 years) in cognitively normal elderly subjects [36]. Similarly, Rauchmann et al. found that baseline plasma p-tau_181_ and NfL were associated with cognitive performance in 5.8 years of follow-up and also predictive of PET Aβ and tau load [37].

Despite their promise, both MRI and plasma biomarkers have their own limitations. Structural MRI is not specific to AD pathology and may be susceptible to non-disease factors, such as developmental factors. On the other hand, blood-based biomarkers only provide a summary measurement and lack spatial specificity. Given the pros and cons, their combination has the potential to yield a better biomarker of disease progression by providing complementary information in prediction, which has not been fully investigated. As MRI is a standard clinical test in the assessment of cognitive impairment and blood draws are easy to obtain, this would offer a particularly appealing approach to prediction. One relevant study by Palmqvist et al. [38] demonstrated that a combination of baseline plasma (p-tau and NfL), MRI-based structural measures, and cognition was predictive of future progression to AD in individuals with subjective cognitive decline and MCI patients. However, there is some circularity in the inclusion of baseline cognitive measures, and they did not investigate the prognostic value of structural MRI and plasma biomarkers alone. In addition, it is also unclear the degree to which these measures would be predictive of cognitive decline in those outside the AD continuum (without evidence of cerebral amyloid). In particular, non-specific measures such as NfL and MRI may allow for the prediction of future neurodegeneration and decline which might reflect normal aging or incipient non-AD neurodegenerative conditions.

In this study, we hypothesize that a combination of structural measurements of MTL subregions extracted from structural MRI and common plasma biomarkers, such as plasma p-tau_181_ and NfL, is predictive of imminent longitudinal disease progression measured by atrophy and cognitive decline, providing a more feasible solution. Since the focus of the current study is on early AD, the analyses were performed in cognitively normal (CN) individuals and MCI patients. In addition, we analyzed both Aβ positive and negative subgroups to investigate the predictive value in subjects outside the AD continuum.

## Methods

### Participants

Longitudinal 3T T1-weighted MRI, longitudinal cognitive measurements, and baseline plasma measures (p-tau_181_ and NfL) of 286 CN and 439 MCI subjects from the Alzheimer’s Disease Neuroimaging Initiative (ADNI) GO and ADNI 2 were included in this study. The two groups were further dichotomized by Aβ status of each participant, determined by thresholding the summary standardized uptake value ratio (SUVR) derived from florbetapir PET^1^ at baseline (available publicly in the processed data on the ADNI website) with a threshold of 1.11 [39]. Details of the ADNI study are provided in Supplementary Material S1. All ethical safeguards and protocols regarding human subjects have been followed.

### Neuroimaging data acquisition and processing

#### Imaging data acquisition

The MRI scans were acquired from different scanners at multiple sites. Up-to-date information about MRI imaging protocols can be found at adni.loni.usc.edu/methods/mri-tool/mri-analysis. For florbetapir PET, images were acquired for 20 min (4 frames of 5-min duration) after a 50-min uptake phase following injection of 10 mCi of tracer. Further detail on the PET acquisition is available at adni.loni.usc.edu/methods/pet-analysis-method/pet-analysis.

#### Baseline MRI measurements of the MTL

Subregions of the MTL, including the anterior/posterior hippocampus, entorhinal cortex (ERC), Brodmann areas (BA) 35 and 36, and parahippocampal cortex (PHC), were automatically segmented in baseline MRI using a tailored pipeline [40, 41], *automatic segmentation of hippocampal subfields-T1* (ASHS-T1)^2^, that overcomes crucial limitations of conventional approaches that are often optimized for whole-brain analysis. Volume measures of the anterior/posterior hippocampus were directly computed from the automatic segmentation. Thickness measurements of the MTL cortical subregions (ERC, BA35, BA36, and PHC) were extracted by applying a graph-based multi-template thickness analysis pipeline [42, 43] to the automatic segmentation. In addition, intracranial volume (ICV) was obtained from the structural MRI using an in-house segmentation software together with ASHS as described in [40].

#### Longitudinal structural MRI marker of disease progression

In our prior study [44], we found that annualized volume change of BA35 is among the best measures in discriminating patients in early phases of AD (including preclinical and early prodromal AD) from Aβ− controls. Therefore, we used this measurement, extracted using the same pipeline in [44], as a proxy of progressive neurodegeneration in this study. In brief, the *Automatic Longitudinal Hippocampal Atrophy* (ALOHA) software [45] was first applied to all pairs of baseline follow-up longitudinal MRI scans with the baseline BA35 segmentation to unbiasedly estimate the BA35 volume of all subsequent MRI scans. Then, a linear model was fitted to the BA35 volume measures of each subject to derive BA35 volume atrophy rate, which was then divided with the baseline BA35 volume to generate a relative volume atrophy rate (in %/year). Bilateral measurements were averaged to increase reliability. We considered longitudinal MRI scans within a 5-year follow-up in this study.

#### Quality control

Comprehensive quality control (details in Supplementary Material S2) was performed to ensure the quality of the baseline and longitudinal BA35 volume change rate measurements. In total, 12 CN and 19 MCI subjects were excluded from the analysis.

### Longitudinal cognitive data processing

To assess cognitive decline, an important marker of disease progression, we included publicly available longitudinal data of an ADNI summary memory measure [ADNI-MEM, which integrates data from the Rey Auditory Verbal Learning Test (RAVLT), AD Assessment Schedule - Cognition (ADAS-Cog), Mini-Mental State Examination (MMSE), and Logical Memory data, details in [46] and the Clinical Dementia Rating Scale Sum of Boxes (CDR-SOB). ADNI-MEM was chosen as a measure of cognitive decline because the current study focuses on the MTL and the early phases of AD. Since the logical memory delayed recall (LDEL) score itself (a cognitive test score that is used to compute ADNI-MEM) has been shown to be sensitive to early AD [47], longitudinal LDEL was additionally included as a marker of longitudinal cognitive decline in a supplementary analysis (Supplementary Table S2). The longitudinal cognitive change rate was computed using linear modeling in the same manner as the measurement of BA35 volume change (the “Longitudinal structural MRI marker of disease progression” section). Different from BA35 volume change, which is a relative change measure (in %/year), the absolute change rate was computed for cognitive measures as no improvement was found in discriminating disease groups when using relative measurements in our prior study [44]. Consistent with the longitudinal MRI measurements, data points in a 5-year follow-up were used to compute longitudinal cognitive change.

To be noted, longitudinal cognitive and structural MRI markers extracted above were used in the receiver operating characteristic (ROC) and univariate analyses (Supplementary Material S3) but were not used in stepwise linear mixed effect modeling (where raw longitudinal measurements were used). Details of the statistical analyses are provided in the “Statistical analysis” section.

In addition, since structural atrophy and cognitive decline within a year may be too small to detect in early phases, longitudinal change measures of participants who had no measurements after 1-year follow-up would likely not be reliable. Therefore, subjects that only have data points within 1-year follow-up (including 1-year follow-up) for all the three longitudinal measurements (BA35, ADNI-MEM, and CDR-SOB) were excluded (additional 24 CN and 58 MCI subjects).

### Plasma p-tau_181_ and NfL data processing

Plasma p-tau_181_ and NfL data are publicly available from the ADNI database. Details of the acquisition and analysis are available at the ADNI website, http://adni.loni.usc.edu. Extreme outliers that were more than six standard deviations (SD) from the mean of the whole study population were identified and excluded from the analysis (plasma p-tau_181_: 2 CN; plasma NfL: 1 CN and 1 MCI).

### Statistical analysis

In total, 41 CN and 78 MCI subjects were excluded, leaving 245 CN and 361 MCI in the final analysis. All statistical tests were two-sided and were conducted in R (http://www.r-project.org). In each diagnostic group (CN and MCI) and the corresponding Aβ+/Aβ− subgroups, stepwise linear mixed effect modeling (lme4 package in R) was performed to identify the subset of baseline structural MRI and plasma measurements that yields the optimal model in predicting the longitudinal change of BA35 volume, ADNI-MEM, and CDR-SOB. In this analysis, raw longitudinal measurements were used. In particular, the modeling consisted of the following steps:

1. A base linear mixed effect model was fitted with baseline age, sex, education, APOE ɛ4 status (carrier or non-carrier), and ICV as fixed effects and with a random intercept and a random slope for the time from baseline for each subject as random effects.
2. Baseline structural MRI and plasma measurements were added to the model iteratively with only one of them added in each iteration: In each iteration, each one of the remaining baseline measurements was added separately to the previous model derived from the last iteration (an interaction term of the one baseline measure with time from baseline was added). The one baseline measurement that yielded the most significant improvement in terms of the Akaike Information Criterion (AIC) was permanently added to the current model, which is the current best model for the next iteration. The selected one baseline measurement was removed from the pool of remaining candidate measures for the next iteration.
3. If none of the remaining baseline measurements significantly improved the model derived from the previous iteration, the iterative process was stopped, and the current best model was considered the final best model.

Before inputting to the model in each analysis, each baseline measurement was standardized by subtracting the mean and dividing by the standard deviation of the subjects in the corresponding analysis. In total, 15 final models were generated: 6 models for BA35 volume change (one of each group of all CN, Aβ− CN, Aβ+ CN, all MCI, Aβ− MCI, and Aβ+ MCI), 6 models for ADNI-MEM change, and 3 models for CDR-SOB change (one for each group of all MCI, Aβ− MCI, and Aβ+ MCI). Analyses were not performed for the all CN, Aβ− CN, and Aβ+ CN groups for CDR-SOB change as the measurements of more than half of the subjects remained unchanged in the 5-year follow-up time.

In addition, to investigate the power of the selected baseline measurements in discriminating the fast and the slow progressors (defined by the first and last terciles in each group/subgroup using the longitudinal measurements in the “Longitudinal structural MRI marker of disease progression” and “Longitudinal cognitive data processing” sections), logistic regression analyses were performed with a binary label of fast/slow progressors as a dependent variable; the selected biomarkers in the corresponding final best models as independent variables; and age, sex, education, APOE ɛ4 status, ICV as covariates. Then, for each model, the receiver operating characteristic (ROC) analysis was performed, and the area under the curve (AUC) was reported (full model). For comparisons, each of these analyses was repeated with the following model settings: (1) model with only covariates (demographic, ICV, and APOE ɛ4 status), referred to as the base model; (2) model with the selected baseline plasma measures identified in the corresponding stepwise mixed effect modeling added on top of the base model, referred to as plasma model; and (3) model with the selected baseline structural MRI measures added on top of the base model, referred to as the MRI model.

For completeness, univariate analysis (partial correlation between each baseline and each longitudinal measurements, with the same set of covariates) was performed to investigate the predictive value of each baseline measurement to disease progression, summarized in Supplementary Material S3.

### Standard protocol approvals, registrations, and patient consents

All research activities were approved by the Institutional Review Boards (IRB) at the participating study sites. Participants provided written informed consent.

## Results

**Fig. 1:**
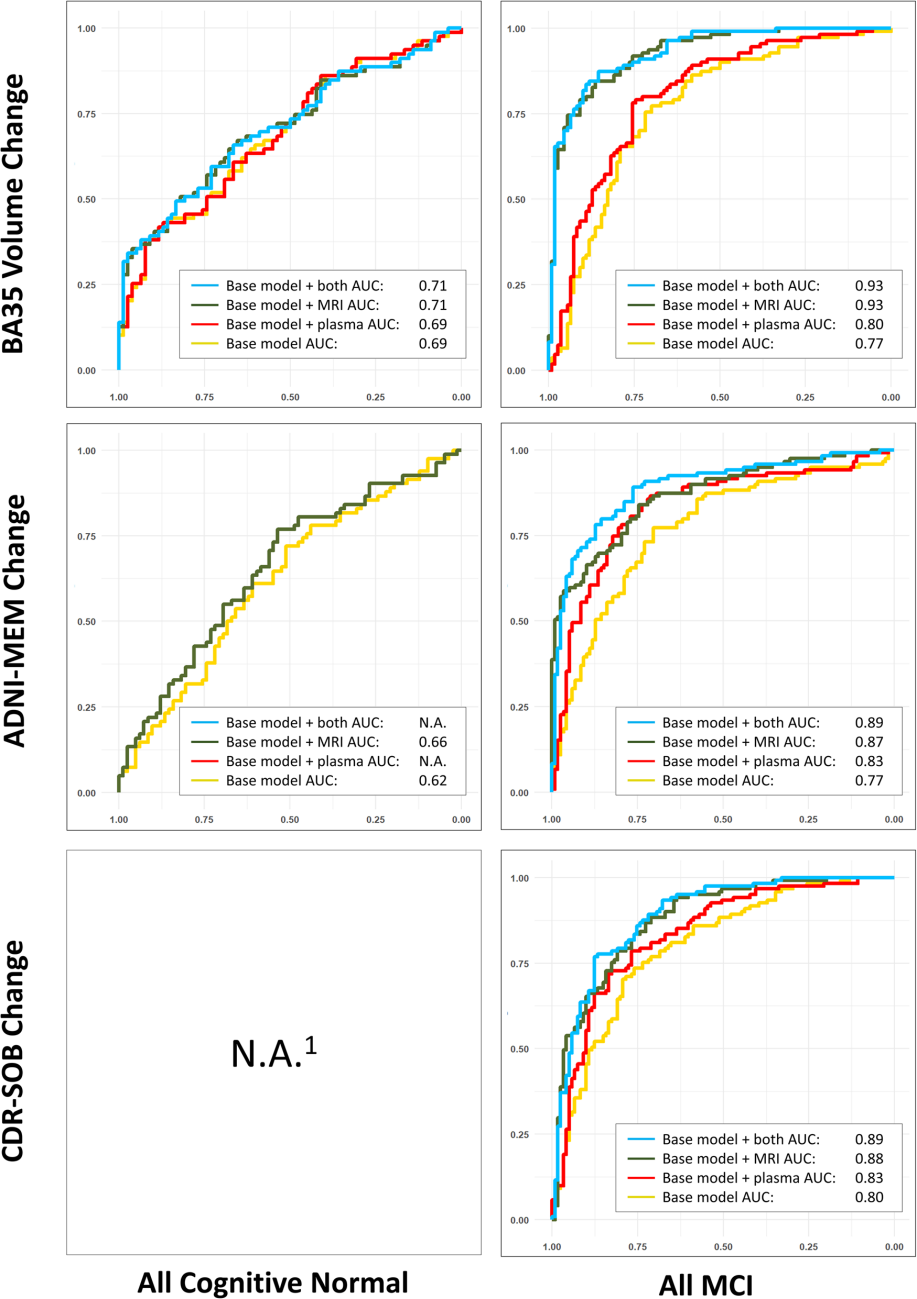
Receiver-operating characteristic (ROC) analyses results of models using both (light blue), either (dark green and red), and none (yellow) of the (baseline model) baseline structural MRI and plasma measurements in the all CN and all MCI groups. Subplots that have less than four lines indicate the corresponding final model did not select both the baseline structural MRI and plasma measurements. *Abbreviations*: MCI, mild cognitive impairment; CDR-SOB, Clinical Dementia Rating Sum-of-Boxes; BA35, Brodmann area 35; AUC, area under the curve; ADNI-MEM, ADNI summary memory scores. ^1^Since the CDR-SOB longitudinal change in most of the all CN subjects (148 out of 248) is equal to 0.0/year, performing linear mixed effect modeling is not appropriate

**Fig. 2:**
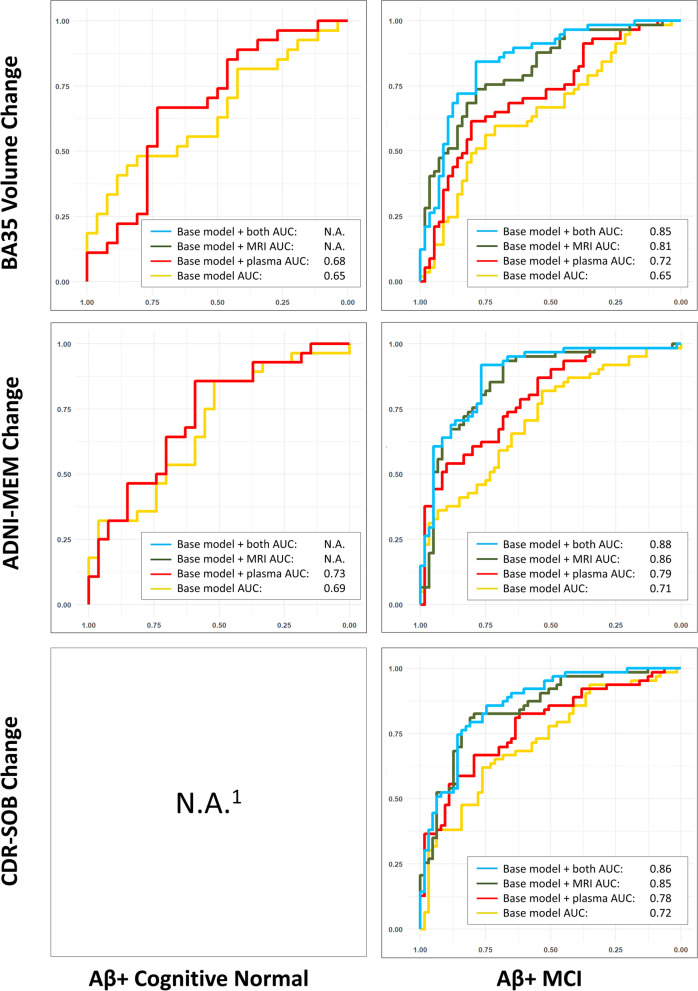
Receiver-operating characteristic (ROC) analysis results of models using both (light blue), either (dark green and red), and none (yellow) of the (baseline model) baseline structural MRI and plasma measurements in the Aβ+ CN and Aβ+ MCI subgroups. Subplots that have less than four lines indicate the corresponding final model did not select both the baseline structural MRI and plasma measurements. *Abbreviations*: MCI, mild cognitive impairment; CDR-SOB, Clinical Dementia Rating Sum-of-Boxes; BA35, Brodmann area 35; AUC, area under the curve; ADNI-MEM, ADNI summary memory scores. ^1^Since the CDR-SOB longitudinal change in most of the Aβ+ CN subjects (39 out of 85) is equal to 0.0/year, performing linear mixed effect modeling is not appropriate

**Fig. 3:**
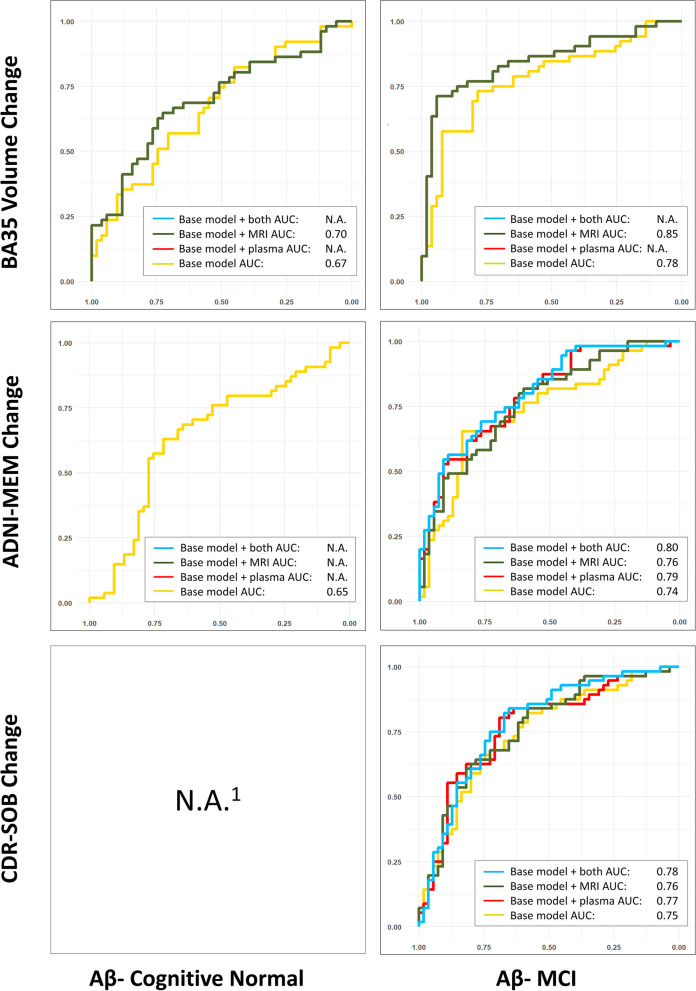
Receiver-operating characteristic (ROC) analyses results of models using both (light blue), either (dark green and red), and none (yellow) of the (baseline model) baseline structural MRI and plasma measurements in the Aβ− CN and Aβ− MCI subgroups. Subplots that have less than four lines indicate the corresponding final model did not select both the baseline structural MRI and plasma measurements. *Abbreviations*: MCI, mild cognitive impairment; CDR-SOB, Clinical Dementia Rating Sum-of-Boxes; BA35, Brodmann area 35; AUC, area under the curve; ADNI-MEM, ADNI summary memory scores. ^1^Since the CDR-SOB longitudinal change in most of the Aβ− CN subjects (108 out of 161) is equal to 0.0 /year, performing linear mixed effect modeling is not appropriate

The characteristics of the remaining subjects (606 in total) of each group/subgroup at baseline are summarized in Table 1. The results of stepwise linear mixed effect modeling and the ROC analysis are summarized in Table 2 and Figs. 1, 2, and 3 and described in detail below.

**Table 1:** Characteristics of the normal controls (CN) and mild cognitive impairment (MCI) participants as well as the Aβ− and Aβ+ subgroups from the Alzheimer’s Disease Neuroimaging Initiative (ADNI) in this study

	**CN**			**MCI**		
	**Aβ−**	**Aβ+**	**All**	**Aβ−**	**Aβ+**	**All**
Number of subjects	158	85	245	168	191	361
Age (years)	72.2 (6.3)	74.8 (5.7)	73.2 (6.2)	69.7 (7.5)	73.3 (6.8)	71.6 (7.3)
Sex (M/F)	83/75	28/57	113/132	91/77	109/82	201/160
Edu (years)	17.1 (2.3)	16.2 (2.7)	16.8 (2.5)	16.4 (2.4)	16.0 (2.8)	16.2 (2.6)
APOE ɛ4 +/−	35/123	40/45	76/169	41/127	128/63	169/192
MMSE	29.1 (1.3)	29.1 (1.0)	29.0 (1.3)	28.6 (1.4)	27.6 (1.8)	28.1 (1.7)
ADNI-MEM	1.13 (0.60)	0.95 (0.56)	1.06 (0.59)	0.63 (0.62)	0.12 (0.63)	0.35 (0.68)
CDR-SOB	0.04 (0.14)	0.08 (0.20)	0.05 (0.16)	1.29 (0.81)	1.56 (0.92)	1.44 (0.88)
AHippo Vol (mm^3^)	1744 (263)	1668 (245)	1720 (259)	1682 (295)	1620 (285)	1651 (293)
PHippo Vol (mm^3^)	1659 (198)	1624 (178)	1648 (192)	1607 (216)	1505 (220)	1555 (225)
ERC Thk (mm)	2.03 (0.16)	2.01 (0.16)	2.02 (0.16)	2.00 (0.20)	1.97 (0.18)	1.98 (0.19)
BA35 Thk (mm)	2.36 (0.16)	2.31 (0.18)	2.35 (0.17)	2.32 (0.21)	2.26 (0.21)	2.29 (0.21)
BA36 Thk (mm)	2.42 (0.23)	2.41 (0.22)	2.42 (0.23)	2.37 (0.26)	2.36 (0.23)	2.37 (0.24)
PHC Thk (mm)	2.15 (0.12)	2.16 (0.17)	2.15 (0.14)	2.16 (0.16)	2.12 (0.15)	2.14 (0.16)
Plasma p-tau_181_ (pg/ml)	14.9 (11.3)	16.9 (7.4)	15.5 (10.1)	13.4 (8.8)	22.0 (12.8)	17.9 (11.8)
Plasma NfL (pg/ml)	32.4 (13.5)	25.7 (13.2)	33.5 (13.4)	33.2 (16.5)	41.2 (16.4)	37.4 (16.9)
Longitudinal structural MRI date Diff (years)	3.3 (1.1)	3.2 (1.2)	3.3 (1.1)	3.2 (1.1)	2.9 (1.2)	3.1 (1.1)
Longitudinal ADNI-MEM date Diff (years)	3.6 (0.9)	3.5 (1.0)	3.5 (1.0)	3.7 (0.8)	3.4 (1.0)	3.6 (0.9)
Longitudinal CDR-SOB date Diff (years)	3.5 (0.9)	3.5 (0.9)	3.5 (0.9)	3.7 (0.8)	3.5 (0.9)	3.6 (0.8)

*Abbreviations: Aβ−/Aβ+ =* β-amyloid negative/positive, *CN* = cognitive normal controls, *Edu* = years of education, *MCI* = mild cognitive impairment, *MMSE* = Mini-Mental State Examination, *ADNI-MEM* = ADNI summary memory scores, *CDR-SOB* = clinical dementia rating sum-of-boxes, *Diff* = difference, *APOE ɛ4 +/−* = APOE ɛ4 gene carrier/non-carrier, *AHippo/PHippo* = anterior/posterior hippocamus, *ERC* = entorhinal cortex, *BA35/BA36* = Brodmann area 35/36, *PHC* = parahippocampal cortex, *Vol* = volume, *Thk* = thickness, *NfL* = neurofilament light chain, *p-tau* = phosphorylated tau.

**Table 2:**
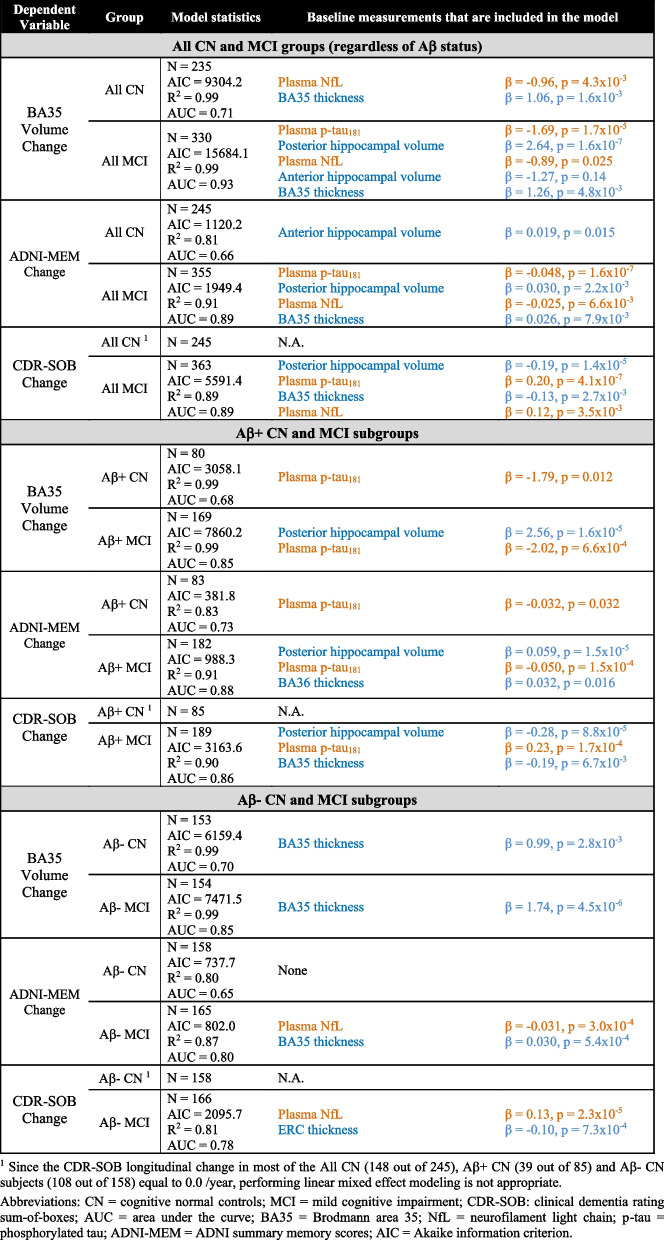
Results of the stepwise linear mixed effect modeling analyses in the all CN and all MCI groups (top), together with Aβ+ (middle) and Aβ− (bottom) subgroups. Variables that were fixed in the model: age, sex, education, intracranial volume, and APOE ɛ4 status. Variables to be selected: baseline structural MRI measurements (highlighted in blue) and baseline plasma measurements (NfL and p-tau_181_, highlighted in orange)

### Significant baseline predictors

In the all CN or all MCI groups, as shown in Table 2 (top), both baseline plasma and structural MRI biomarkers were consistently selected by almost all the models (except for predicting ADNI-MEM change in the all CN group). Similar results, albeit with some differences in the selected predictors, were observed in the Aβ+ MCI subgroup (Table 2, middle). Alternatively, in the Aβ+ CN group, baseline plasma p-tau_181_ alone was selected. In the Aβ− subgroups (Table 2, bottom), structural MRI measures were selected in 4 out of 5 models, and plasma NfL, a measure of neurodegeneration, was included in 2 out of the 5 models. Neither plasma nor MRI measures provided additional information in predicting ADNI-MEM change in Aβ− CN. We note that structural MRI measurements were included in most of the models, and BA35 thickness was the most commonly selected measurement (was included in 8 out of 15 final models). Importantly, when comparing the selected plasma measures in the Aβ+ and Aβ− subgroups, plasma p-tau_181_ was only selected in the Aβ+ subgroups, and plasma NfL was only selected in the Aβ− ones. The results of LDEL in Supplementary Table S2 were similar to that of ADNI-MEM with a difference in the Aβ− CN group, in which parahippocampal cortex thickness was selected in the final model for LDEL while none of the baseline measurements was included for ADNI-MEM.

### Distinguishing the fast and slow progressors

The ROC curves in Fig. 1 consistently demonstrate that the combination of both the selected baseline plasma and structural MRI measurements have the largest AUC (in absolute terms) in identifying the fast from the slow progressors (the first and last terciles of each longitudinal measurement) compared to models using either of these biomarkers alone, as well as base models with only demographic, ICV, and APOE ɛ4 information. As expected, the AUCs in the MCI groups are larger than those in the CN groups. The results are similar in the Aβ− and Aβ+ subgroups (Figs. 2 and 3).

## Discussion

The aim of this study was to test the hypothesis that a combination of cross-sectional structural MRI and plasma biomarkers would be predictive of early AD-associated near-term disease progression, which would fall within the timeframe of potential clinical trials. The present findings demonstrated that baseline plasma and structural MRI biomarkers provided complementary information in predicting longitudinal atrophy and cognitive decline in controls and MCI (Table 2, Fig. 1). These relationships were maintained, albeit with some differences in selected predictors, when limited to Aβ+ (preclinical and prodromal AD; Table 2, middle Fig. 2). However, in the Aβ− subgroups (Table 2, bottom; Fig. 3), plasma measures were only included in two models (NfL in predicting ADNI-MEM and CDR-SOB in Aβ− MCI), but most included structural measures as well. This finding is consistent with the notion that non-specific measures of neurodegeneration (MRI, plasma NfL), may be sensitive to non-AD related longitudinal change in the brain structure and cognition while more AD-specific measures (plasma p-tau_181_) are predictive of progression within the AD continuum. In addition, ROC analysis of each model showed that the proposed biomarkers were able to discriminate fast and slow progressors (the first and last terciles) with AUCs ranging from 0.78 to 0.93 in MCI and 0.65 to 0.73 in CN.

### Predicting disease progression with baseline plasma and structural MRI biomarkers

Baseline plasma and structural MRI biomarkers were both included in the majority of the models (9 out of 15 final stepwise linear mixed effect models) supporting the hypothesis that molecular and structural MRI biomarkers provide complementary information in predicting imminent disease progression, which, in part, is consistent with that shown in Palmqvist et al. [38] This phenomenon was more commonly observed in models associated with MCI (8 out of 9 final models). When targeting CN, plasma and structural MRI measures were both included when considering the entire group, again supporting a complementary nature. However, in the preclinical phase (Aβ+ CN), only the p-tau_181_ plasma measure was predictive of progression while for age-related decline (Aβ− CN), only a structural measure provided additional prediction beyond the base model.

Plasma p-tau_181_ was commonly included in models for the whole cohort and the Aβ+ subgroups, but not included in models for Aβ− ones. This is consistent with the fact that plasma p-tau_181_ is the only AD-specific biomarker included in these analyses. As neurofibrillary tangle pathology is the primary driver of neurodegeneration and cognitive decline in the AD continuum, it is not surprising that p-tau levels (likely related to NFT burden) would be predictive of decline in those with likely AD pathology (Aβ+). Structural MRI and plasma NfL measure brain injury that could be due to a number of non-AD neurodegenerative conditions and even “normal” brain aging and, thus, may be better in predicting further atrophy and cognitive decline in Aβ− subjects.

The results of this study are consistent with prior work showing that combinations of biomarkers including structural MRI and CSF or PET provide better prediction than these measures alone in both MCI [2–6] and CN [7–10, 12] cohorts. However, a combination of structural MRI and plasma biomarkers, rather than CSF-based ones, is more feasible in clinical trials and particularly clinical practice, as plasma biomarkers are non-invasive and less expensive.

### Effectiveness in identifying fast and slow progressors

Enriching cohorts with at-risk individuals is crucial for clinical trials targeting early AD, especially in the preclinical phase. Hence, biomarkers that can identify fast and slow progressors will play a significant role in future drug and treatment development. The results shown in Figs. 1 and 2 demonstrate that the proposed combined plasma and structural MRI biomarker achieved 0.89–0.93 AUCs in discriminating fast (first tercile) and slow (last tercile) progressors in the all MCI cohort and 0.85–0.88 AUCs in Aβ+ MCI, providing a reliable criterion. In all CN and Aβ+ CN (preclinical AD), the AUC values were more modest (0.66–0.71 and 0.68–0.73, respectively) but indicate the proposed biomarker(s) would provide meaningful benefit in identifying high-risk cognitively unimpaired individuals. In addition, the combined biomarkers consistently outperformed the individual ones in most tasks (in absolute terms, Figs. 1, 2, and 3), which echoes the claim about the two types of biomarkers being complementary.

### Potential use of structural MRI biomarkers in predicting normal aging-related decline

From the results of Aβ− CN (Table 2 bottom), i.e., normal aging, we observed baseline BA35 thickness significantly predicted longitudinal BA35 atrophy (*β* = 0.99, *p* = 2.8 × 10^−3^). Although none of the baseline measures was selected in the final model when predicting ADNI-MEM (Table 2 bottom), we did find, in a supplementary analysis (Supplementary Table S2 bottom), parahippocampal cortex thickness was predictive of the decline of logical memory delayed recall score, a cognitive memory test score that has been shown to be sensitive to early AD [47]. These results indicate that our structural MRI biomarkers, generated using a tailored pipeline, are predictive of brain atrophy and may be sensitive to cognitive decline not only in the AD continuum but also in presumably “normal” aging. However, it is worth noting that one driver of disease progression in non-AD (Aβ−) CN adults may be primary age-related tauopathy, or PART, in which neurofibrillary tangles accumulate in the absence of amyloid [11]. So, it would at least be conceivable that plasma p-tau_181_ might be related to BA35 atrophy (as it is the first region of NFTs) and cognitive decline in amyloid-negative individuals, as has been observed with both CSF p-tau_181_ [48–51] and tau PET studies. This may suggest a reduced sensitivity of plasma p-tau to PART. Regardless, the proposed structural MRI biomarker may identify older adults that are more or less likely to suffer significant age-associated decline (Fig. 3), providing a potential marker for future studies on normal aging and super-agers.

### Limitations and future work

There are several limitations in this study. First, only two plasma measurements, i.e., plasma p-tau_181_ and NfL, were included in the current analyses. In future work, adding in other promising plasma measures, such as plasma p-tau_217_ [16], which may be more sensitive to earlier AD pathology than p-tau_181_, and glial fibrillary acidic protein may further increase the predictive power of the combined biomarker. Second, although structural MRI measurements were consistently included in most models, the most predictive measures varied (BA35 thickness 8 times, posterior hippocampal volume 6 times, anterior hippocampal volume 2 times, ERC thickness 1 time, and BA36 thickness 1 time) in different models, making the specificity of the MTL effects difficult to interpret. A summary value derived from all the MTL subregional measurements using event-based modeling may provide a more consistent and sensitive measurement, which will be investigated in future work. Additionally, future work will need to validate these findings in other independent datasets. Nonetheless, the present data support the notion that plasma and structural MRI biomarkers provide a prediction of the rate of future cognitive and neurodegenerative progression that may be particularly useful in clinical trial stratification and prognosis.

### Supplementary Information

**Additional file 1:** **S.1.** Alzheimer’s Disease Neuroimaging Initiative (ADNI) study. **S.2.** Quality control of MRI image processing. **S.3.** Univariate analysis between baseline and longitudinal measurements. **Table S1.** Partial correlation, controlling for age, sex, education, APOE ɛ4 status and intracranial volume, between each baseline structural MRI and plasma biomarker and each longitudinal measurement. Correlations with p value less than 0.05 are highlighted in red background. **Fig. S1.** Scatter plots of baseline posterior hippocampal volume and all longitudinal measurements, corrected for age, sex, education, APOE ɛ4 status and intracranial volume. Abbreviations: CN = cognitive normal controls; MCI = mild cognitive impairment; CDR-SOB: clinical dementia rating sum-of-boxes; ADNI-MEM = ADNI summary memory score; BA35 = Brodmann area 35. **Fig. S2.** Scatter plots of baseline BA35 thickness and all longitudinal measurements, corrected for age, sex, education, APOE ɛ4 status and intracranial volume. Abbreviations: CN = cognitive normal controls; MCI = mild cognitive impairment; CDR-SOB: clinical dementia rating sum-of-boxes; ADNI-MEM = ADNI summary memory score; BA35 = Brodmann area 35. **Fig. S3.** Scatter plots of baseline plasma NfL and all longitudinal measurements, corrected for age, sex, education, APOE ɛ4 status and intracranial volume. Abbreviations: CN = cognitive normal controls; MCI = mild cognitive impairment; CDR-SOB: clinical dementia rating sum-of-boxes; ADNI-MEM = ADNI summary memory score; BA35 = Brodmann area 35; NfL = neurofilament light chain. **Fig. S4.** Scatter plots of baseline plasma p-tau181 and all longitudinal measurements, corrected for age, sex, education, APOE ɛ4 status and intracranial volume. Abbreviations: CN = cognitive normal controls; MCI = mild cognitive impairment; CDR-SOB: clinical dementia rating sum-of-boxes; ADNI-MEM = ADNI summary memory score; BA35 = Brodmann area 35; p-tau = phosphorylated tau. **Table S2.** Results of the stepwise linear mixed effect modeling analyses for logical memory delayed recall (LDEL) in the All CN and All MCI groups (top), together with Aβ+ (middle) and Aβ- (bottom) subgroups. Variables that were fixed in the model: age, sex, education, intracranial volume and APOE ɛ4 status. Variables to be selected: baseline structural MRI measurements (highlighted in blue) and baseline plasma measurements (NfL and p-tau181, highlighted in orange).

## Data Availability

All raw data used in this study are publicly available and granted access by the relevant study’s ADNI review committee. Processed data not provided in the article because of space limitations may be shared at the request of any qualified investigator for purposes of replicating procedures and results.

## Footnotes

1 Full description of florbetapir PET can be found at this website: adni.bitbucket.io/reference/docs/UCBERKELEYAV45/ADNI_AV45_Methods_JagustLab_06.25.15.pdf

2 https://sites.google.com/view/ashs-dox/
