# Correction: Baseline structural MRI and plasma biomarkers predict longitudinal structural atrophy and cognitive decline in early Alzheimer’s disease

**DOI:** 10.1186/s13195-023-01374-8

**Published:** 2024-01-12

**Authors:** Long Xie, Sandhitsu R. Das, Laura E. M. Wisse, Ranjit Ittyerah, Robin de Flores, Leslie M. Shaw, Paul A. Yushkevich, David A. Wolk, for the Alzheimer’s Disease Neuroimaging Initiative

**Affiliations:** 1 Penn Image Computing and Science Laboratory (PICSL), Department of Radiology, University of Pennsylvania, 3700 Hamilton Walk, Suite D600, Richards Building 6 Floor, Philadelphia, PA 19104 USA; 2 Penn Memory Center, University of Pennsylvania, Philadelphia, PA USA; 3 Department of Diagnostic Radiology, Lund University, Lund, Sweden; 4 Department of Pathology and Laboratory Medicine, University of Pennsylvania, Philadelphia, PA USA

**Correction: Alz Res Therapy 15, 79 (2023)**

**https://doi.org/10.1186/s13195-023-01210-z**

Following the publication of the original article [1], the author reported that “Additional file 1” file in the published article is not the correct file.

The original article [1] has been updated.
