# Supplementary material for: Baseline structural MRI and plasma biomarkers predict longitudinal structural atrophy and cognitive decline in early Alzheimer’s disease
Source: Alzheimers Res Ther. 2023 Apr 11;15:79. doi: 10.1186/s13195-023-01210-z (PMC10088234; doi:10.1186/s13195-023-01210-z)
Supplement: Supplementary file 1 — Additional file 1: S.1. Alzheimer’s Disease Neuroimaging Initiative (ADNI) study. S.2. Quality control of MRI image processing. S.3. Univariate analysis between baseline and longitudinal measurements. Table S1. Partial correlation, controlling for age, sex, education, APOE ɛ4 status and intracranial volume, between each baseline structural MRI and plasma biomarker and each longitudinal measurement. Correlations with p value less than 0.05 are highlighted in red background. Fig. S1. Scatter plots of baseline posterior hippocampal volume and all longitudinal measurements, corrected for age, sex, education, APOE ɛ4 status and intracranial volume. Abbreviations: CN = cognitive normal controls; MCI = mild cognitive impairment; CDR-SOB: clinical dementia rating sum-of-boxes; ADNI-MEM = ADNI summary memory score; BA35 = Brodmann area 35. Fig. S2. Scatter plots of baseline BA35 thickness and all longitudinal measurements, corrected for age, sex, education, APOE ɛ4 status and intracranial volume. Abbreviations: CN = cognitive normal controls; MCI = mild cognitive impairment; CDR-SOB: clinical dementia rating sum-of-boxes; ADNI-MEM = ADNI summary memory score; BA35 = Brodmann area 35. Fig. S3. Scatter plots of baseline plasma NfL and all longitudinal measurements, corrected for age, sex, education, APOE ɛ4 status and intracranial volume. Abbreviations: CN = cognitive normal controls; MCI = mild cognitive impairment; CDR-SOB: clinical dementia rating sum-of-boxes; ADNI-MEM = ADNI summary memory score; BA35 = Brodmann area 35; NfL = neurofilament light chain. Fig. S4. Scatter plots of baseline plasma p-tau181 and all longitudinal measurements, corrected for age, sex, education, APOE ɛ4 status and intracranial volume. Abbreviations: CN = cognitive normal controls; MCI = mild cognitive impairment; CDR-SOB: clinical dementia rating sum-of-boxes; ADNI-MEM = ADNI summary memory score; BA35 = Brodmann area 35; p-tau = phosphorylated tau. Table S2. Results of the stepwise linear mixed [file 13195_2023_1210_MOESM1_ESM.docx]

**Supplementary material**

**S.1 Alzheimer’s Disease Neuroimaging Initiative (ADNI) study**

Data used in preparation of this article were obtained from the Alzheimer’s Disease Neuroimaging Initiative (ADNI) database (adni.loni.usc.edu). As such, the investigators within the ADNI contributed to the design and implementation of ADNI and/or provided data but did not participate in analysis or writing of this report. A complete listing of ADNI investigators can be found at: <http://adni.loni.usc.edu/wp-content/uploads/how_to_apply/ADNI_Acknowledgement_List.pdf>. The ADNI was launched in 2003 as a public-private partnership, led by Principal Investigator Michael W. Weiner, MD. The primary goal of ADNI has been to test whether serial magnetic resonance imaging (MRI), positron emission tomography (PET), other biological markers, and clinical and neuropsychological assessment can be combined to measure the progression of mild cognitive impairment and early Alzheimer’s disease. For up-to-date information, see www.adni-info.org.

**S.2 Quality control of MRI image processing**

Comprehensive quality control was performed to ensure the quality of the baseline and longitudinal BA35 volume change rate measurements. First, baseline MRI scans of all the subjects were visually checked to exclude subjects with severe motion artifact or blurring. Second, visual inspection was also performed on the baseline ASHS-T1 segmentations and small errors in a subset of individuals were identified. Third, the quality of longitudinal estimation was assessed by checking the alignment between baseline and follow-up MRI scans. Since it is not feasible to look at all registration pairs, an effective sampling strategy described in Xie et al. (Human brain mapping, 2020) was used to select a subset of pairs for inspection. Pairs that were marked failed were not used in the longitudinal change rate computation.

**S.3 Univariate analysis between baseline and longitudinal measurements**

Supplementary Table S1 shows results of partial correlation analysis between each baseline MRI or plasma biomarker and each prospective longitudinal measurements (BA35 change rate, ADNI-MEM change rate, CDR-SOB change rate), controlling for age, sex, education, APOE ɛ4 status and intracranial volume. Scatter plots of the most selected baseline structural MRI [posterior hippocampal volume (Supplementary Figure S1) and BA35 thickness (Supplementary Figure S2)] and plasma [NfL (Supplementary Figure S3) and p-tau_181_ (Supplementary Figure S4)] measurements are shown in the corresponding figures.

**Supplementary Table S1.** Partial correlation, controlling for age, sex, education, APOE ɛ4 status and intracranial volume, between each baseline structural MRI and plasma biomarker and each longitudinal measurement. Correlations with *p* value less than 0.05 are highlighted in red background.

|  |  | BA35 Change Rate (%/year) | ADNI-MEM Change Rate (/year) | CDR-SOB Change Rate (/year) |
| --- | --- | --- | --- | --- |
| All CN | **AHippo Vol** | *rho* = 0.14, *p* > 0.5 | *rho* = 0.13, *p* > 0.1 | N.A.^1^ |
|  | **PHippo Vol** | *rho* = 0.13, *p* > 0.1 | *rho* = 0.04, *p* > 0.1 |  |
|  | **ERC Thk** | *rho* = 0.03, *p* > 0.1 | *rho* = 0.16, *p* = 0.040 |  |
|  | **BA35 Thk** | *rho* = 0.20, *p* = 0.012 | *rho* = 0.03, *p* > 0.1 |  |
|  | **BA36 Thk** | *rho* = 0.10, *p* > 0.1 | *rho* = 0.00, *p* > 0.1 |  |
|  | **PHC Thk** | *rho* = 0.10, *p* > 0.1 | *rho* = 0.08, *p* > 0.1 |  |
|  | **Plasma NfL** | *rho* = -0.17, *p* = 0.040 | *rho* = -0.12, *p* > 0.1 |  |
|  | **Plasma p-tau_181_** | *rho* = -0.06, *p* > 0.1 | *rho* = -0.11, *p* > 0.1 |  |
| Aβ+ CN | **AHippo Vol** | *rho* = 0.18, *p* > 0.1 | *rho* = 0.25, *p* > 0.05 |  |
|  | **PHippo Vol** | *rho* = 0.10, *p* > 0.1 | *rho* = 0.14, *p* > 0.1 |  |
|  | **ERC Thk** | *rho* = 0.08, *p* > 0.1 | *rho* = 0.27, *p* = 0.045 |  |
|  | **BA35 Thk** | *rho* = 0.14, *p* > 0.1 | *rho* = 0.07, *p* > 0.1 |  |
|  | **BA36 Thk** | *rho* = 0.16, *p* > 0.1 | *rho* = 0.09, *p* > 0.1 |  |
|  | **PHC Thk** | *rho* = 0.03, *p* > 0.1 | *rho* = 0.19, *p* > 0.1 |  |
|  | **Plasma NfL** | *rho* = -0.17, *p* > 0.1 | *rho* = -0.06, *p* > 0.1 |  |
|  | **Plasma p-tau_181_** | *rho* = -0.26, *p* = 0.050 | *rho* = -0.19, *p* > 0.1 |  |
| Aβ- CN | **AHippo Vol** | *rho* = 0.15, *p* > 0.1 | *rho* = 0.11, *p* > 0.1 |  |
|  | **PHippo Vol** | *rho* = 0.27, *p* = 0.0071 | *rho* = 0.00, *p* > 0.1 |  |
|  | **ERC Thk** | *rho* = 0.07, *p* > 0.1 | *rho* = 0.19, *p* = 0.049 |  |
|  | **BA35 Thk** | *rho* = 0.23, *p* = 0.019 | *rho* = 0.07, *p* > 0.1 |  |
|  | **BA36 Thk** | *rho* = 0.14, *p* > 0.1 | *rho* = 0.03, *p* > 0.1 |  |
|  | **PHC Thk** | *rho* = 0.28, *p* = 0.040 | *rho* = 0.11, *p* > 0.1 |  |
|  | **Plasma NfL** | *rho* = -0.04, *p* > 0.1 | *rho* = -0.13, *p* > 0.1 |  |
|  | **Plasma p-tau_181_** | *rho* = 0.09, *p* > 0.1 | *rho* = -0.02, *p* > 0.1 |  |
| All MCI | **AHippo Vol** | *rho* = 0.32, *p* = $1.7\times{10}^{-6}$ | *rho* = 0.21, *p* = 0.0012 | *rho* = -0.24, *p* = $2.2\times{10}^{-4}$ |
|  | **PHippo Vol** | *rho* = 0.51, *p* = $3.8\times{10}^{-16}$ | *rho* = 0.39, *p* = $3.7\times{10}^{-10}$ | *rho* = -0.35, *p* = $1.4\times{10}^{-8}$ |
|  | **ERC Thk** | *rho* = 0.28, *p* = $2.7\times{10}^{-5}$ | *rho* = 0.19, *p* = 0.0033 | *rho* = -0.20, *p* = 0.0011 |
|  | **BA35 Thk** | *rho* = 0.45, *p* = $3.2\times{10}^{-12}$ | *rho* = 0.31, *p* = $9.0\times{10}^{-7}$ | *rho* = -0.34, *p* = $7.4\times{10}^{-8}$ |
|  | **BA36 Thk** | *rho* = 0.13, *p* > 0.05 | *rho* = 0.20, *p* = 0.0018 | *rho* = -0.20, *p* = 0.0020 |
|  | **PHC Thk** | *rho* = 0.24, *p* = $3.0\times{10}^{-4}$ | *rho* = 0.20, *p* = 0.0018 | *rho* = -0.21, *p* = $7.9\times{10}^{-4}$ |
|  | **Plasma NfL** | *rho* = -0.19, *p* = 0.0051 | *rho* = -0.25, *p* = $8.1\times{10}^{-5}$ | *rho* = 0.23, *p* = $2.3\times{10}^{-4}$ |
|  | **Plasma p-tau_181_** | *rho* = -0.29, *p* = $1.4\times{10}^{-5}$ | *rho* = -0.33, *p* = $2.6\times{10}^{-7}$ | *rho* = 0.25, *p* = $7.7\times{10}^{-4}$ |
| Aβ+ MCI | **AHippo Vol** | *rho* = 0.30, *p* = 0.0012 | *rho* = 0.21, *p* = 0.019 | *rho* = -0.33, *p* = $1.8\times{10}^{-4}$ |
|  | **PHippo Vol** | *rho* = 0.50, *p* = $1.9\times{10}^{-8}$ | *rho* = 0.43, *p* = $1.1\times{10}^{-6}$ | *rho* = -0.46, *p* = $4.7\times{10}^{-8}$ |
|  | **ERC Thk** | *rho* = 0.23, *p* = 0.016 | *rho* = 0.19, *p* = 0.038 | *rho* = -0.28, *p* = 0.0012 |
|  | **BA35 Thk** | *rho* = 0.49, *p* = $4.5 \times{10}^{-8}$ | *rho* = 0.32, *p* = $3.8\times{10}^{-4}$ | *rho* = -0.40, *p* = $2.9\times{10}^{-6}$ |
|  | **BA36 Thk** | *rho* = 0.23, *p* = 0.016 | *rho* = 0.30, *p* = $8.5\times{10}^{-4}$ | *rho* = -0.24, *p* = 0.0059 |
|  | **PHC Thk** | *rho* = 0.16, *p* > 0.05 | *rho* = 0.18, *p* = 0.044 | *rho* = -0.23, *p* = 0.0082 |
|  | **Plasma NfL** | *rho* = -0.29, *p* = 0.0015 | *rho* = -0.26, *p* = 0.0039 | *rho* = 0.31, *p* = $4.7\times{10}^{-4}$ |
|  | **Plasma p-tau_181_** | *rho* = -0.28, *p* = 0.0029 | *rho* = -0.33, *p* = $2.7\times{10}^{-4}$ | *rho* = 0.27, *p* = 0.0023 |
| Aβ- MCI | **AHippo Vol** | *rho* = 0.33, *p =* $5.9\times{10}^{-4}$ | *rho* = 0.23, *p* = 0.015 | *rho* = -0.38, *p* = $4.5\times{10}^{-5}$ |
|  | **PHippo Vol** | *rho* = 0.40, *p* = $3.2\times{10}^{-5}$ | *rho* = 0.19, *p* > 0.05 | *rho* = -0.35, *p* = $1.6\times{10}^{-4}$ |
|  | **ERC Thk** | *rho* = 0.29, *p* = 0.0034 | *rho* = 0.27, *p* = 0.0049 | *rho* = -0.35, *p* = $1.5\times{10}^{-4}$ |
|  | **BA35 Thk** | *rho* = 0.43, *p* = $4.4\times{10}^{-6}$ | *rho* = 0.26, *p* = 0.0051 | *rho* = -0.32, *p* = $5.6\times{10}^{-4}$ |
|  | **BA36 Thk** | *rho* = 0.10, *p* > 0.1 | *rho* = 0.18, *p* > 0.05 | *rho* = -0.19, *p* = 0.047 |
|  | **PHC Thk** | *rho* = 0.30, *p* = 0.0020 | *rho* = 0.21, *p* = 0.028 | *rho* = -0.28, *p* = 0.0025 |
|  | **Plasma NfL** | *rho* = -0.15, *p* > 0.1 | *rho* = -0.30, *p* = 0.0012 | *rho* = 0.23, *p* = 0.017 |
|  | **Plasma p-tau_181_** | *rho* = -0.09, *p* > 0.1 | *rho* = 0.01, *p* > 0.1 | *rho* = -0.06, *p* > 0.1 |

^1^ Since the CDR-SOB longitudinal change in most of the CN subjects equals to 0.0 /year, performing partial correlation analysis is not appropriate.

Abbreviations: Aβ-/Aβ+: β-amyloid negative/positive; CN = cognitive normal controls; MCI = mild cognitive impairment; CDR-SOB: clinical dementia rating sum-of-boxes; AUC = area under the curve; BA35 = Brodmann area 35; NfL = neurofilament light chain; p-tau = phosphorylated tau; ADNI-MEM = ADNI summary memory score; AHippo/PHippo = anterior/posterior hippocampus; ERC = entorhinal cortex; BA35/36 = Brodmann area 35/36; PHC = parahippocampal cortex; Vol = volume; Thk = thickness.


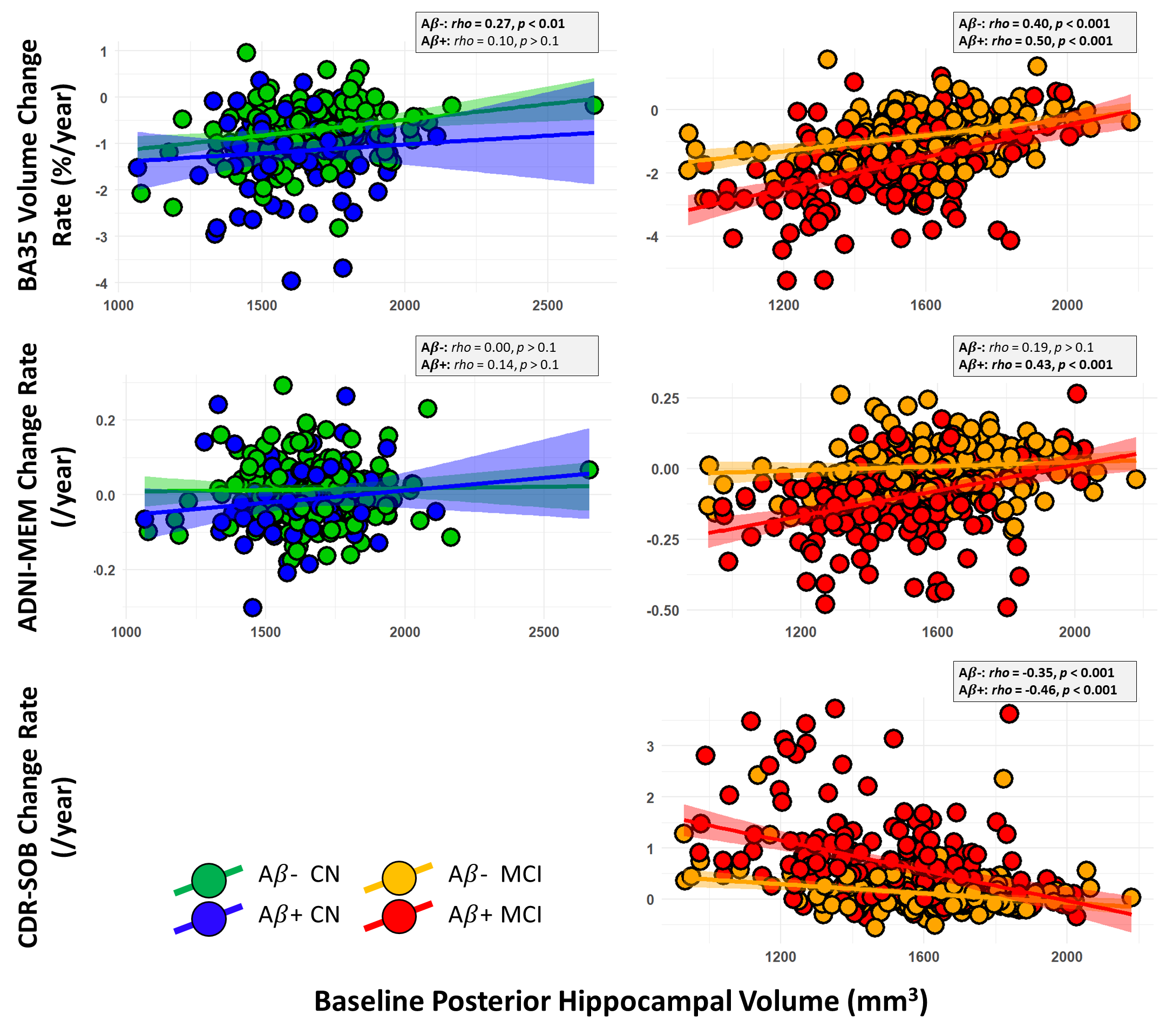


**Supplementary Figure S1.** Scatter plots of baseline posterior hippocampal volume and all longitudinal measurements, corrected for age, sex, education, APOE ɛ4 status and intracranial volume. Abbreviations: CN = cognitive normal controls; MCI = mild cognitive impairment; CDR-SOB: clinical dementia rating sum-of-boxes; ADNI-MEM = ADNI summary memory score; BA35 = Brodmann area 35.


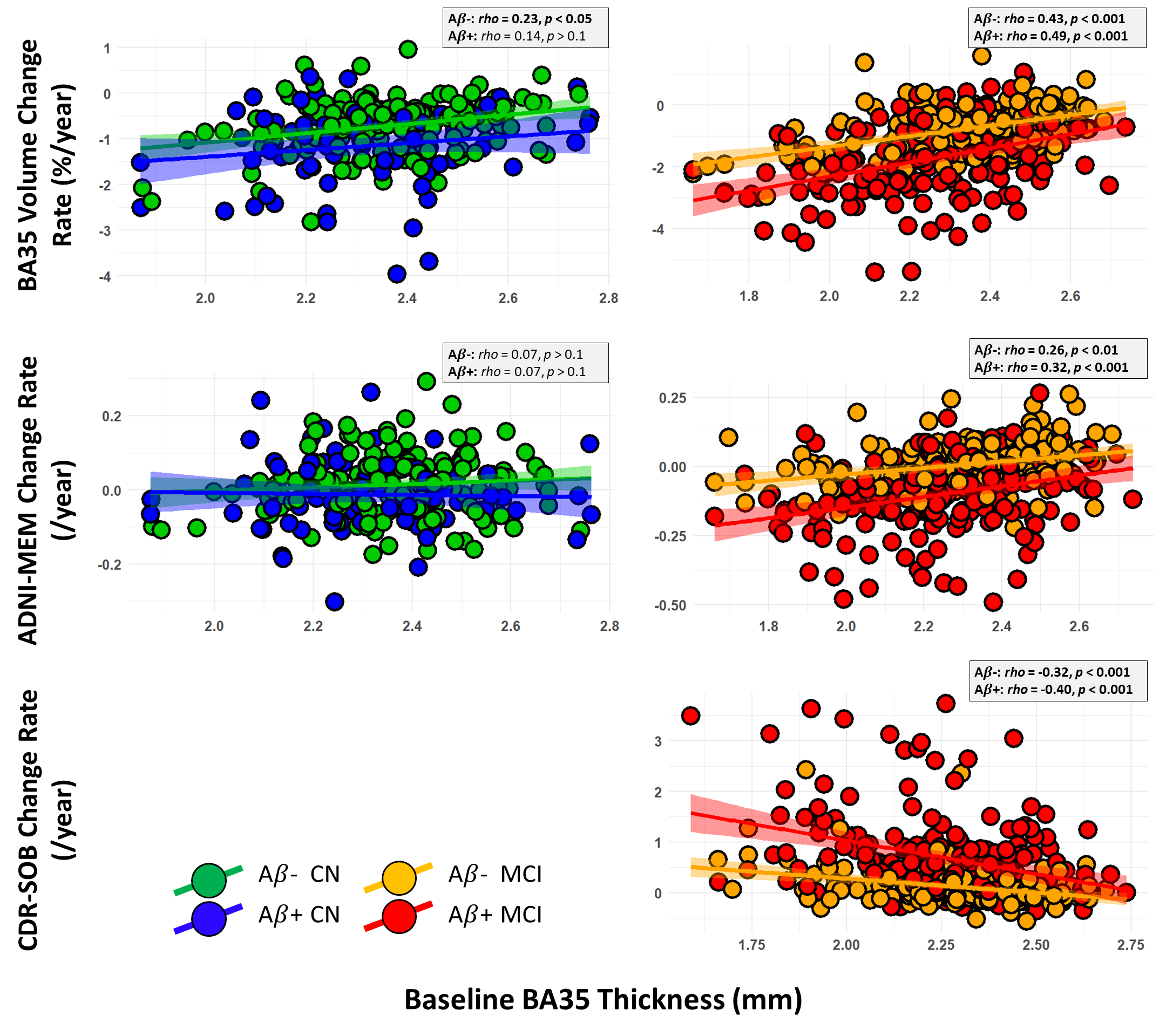


**Supplementary Figure S2.** Scatter plots of baseline BA35 thickness and all longitudinal measurements, corrected for age, sex, education, APOE ɛ4 status and intracranial volume. Abbreviations: CN = cognitive normal controls; MCI = mild cognitive impairment; CDR-SOB: clinical dementia rating sum-of-boxes; ADNI-MEM = ADNI summary memory score; BA35 = Brodmann area 35.


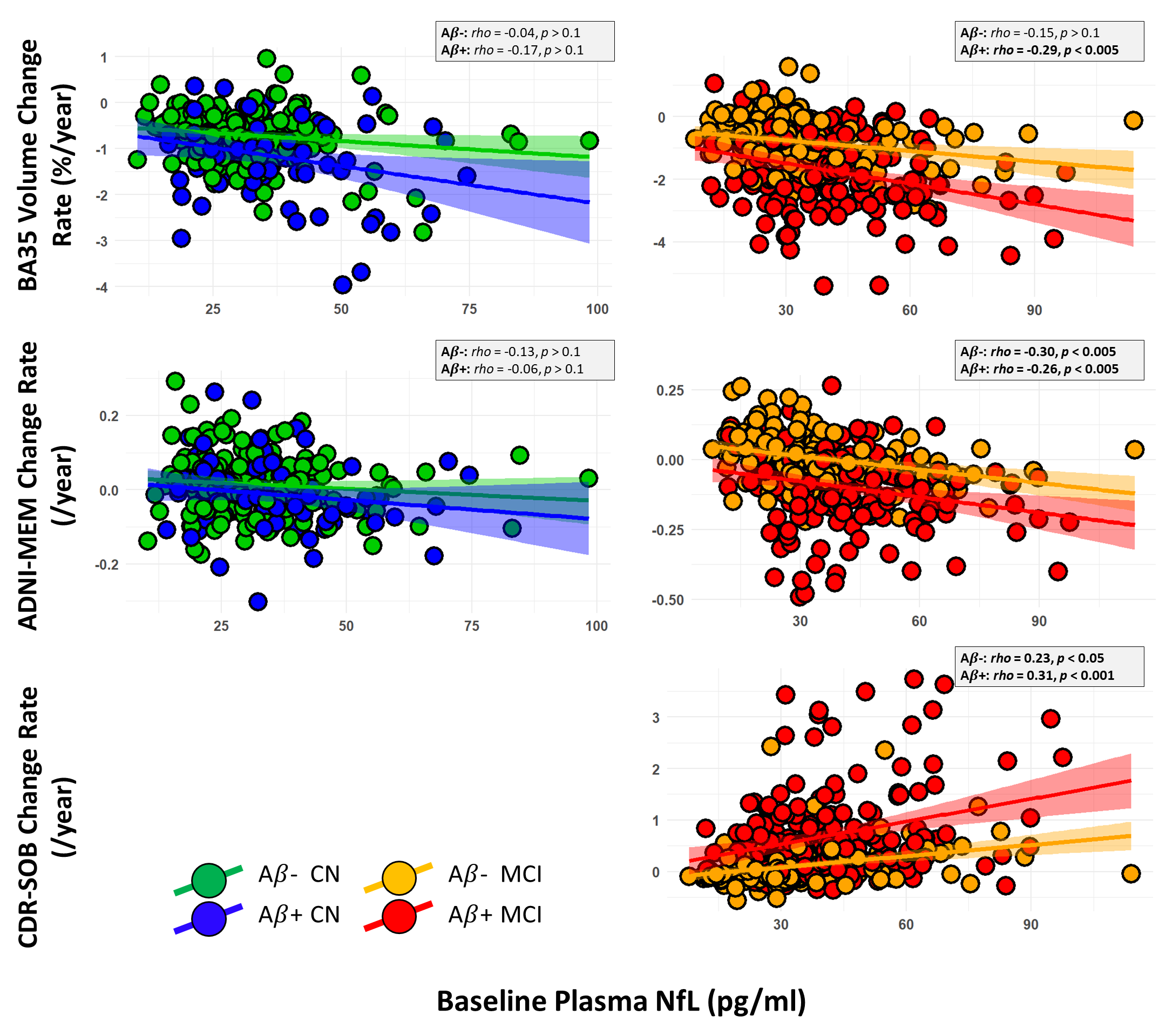


**Supplementary Figure S3.** Scatter plots of baseline plasma NfL and all longitudinal measurements, corrected for age, sex, education, APOE ɛ4 status and intracranial volume. Abbreviations: CN = cognitive normal controls; MCI = mild cognitive impairment; CDR-SOB: clinical dementia rating sum-of-boxes; ADNI-MEM = ADNI summary memory score; BA35 = Brodmann area 35; NfL = neurofilament light chain.


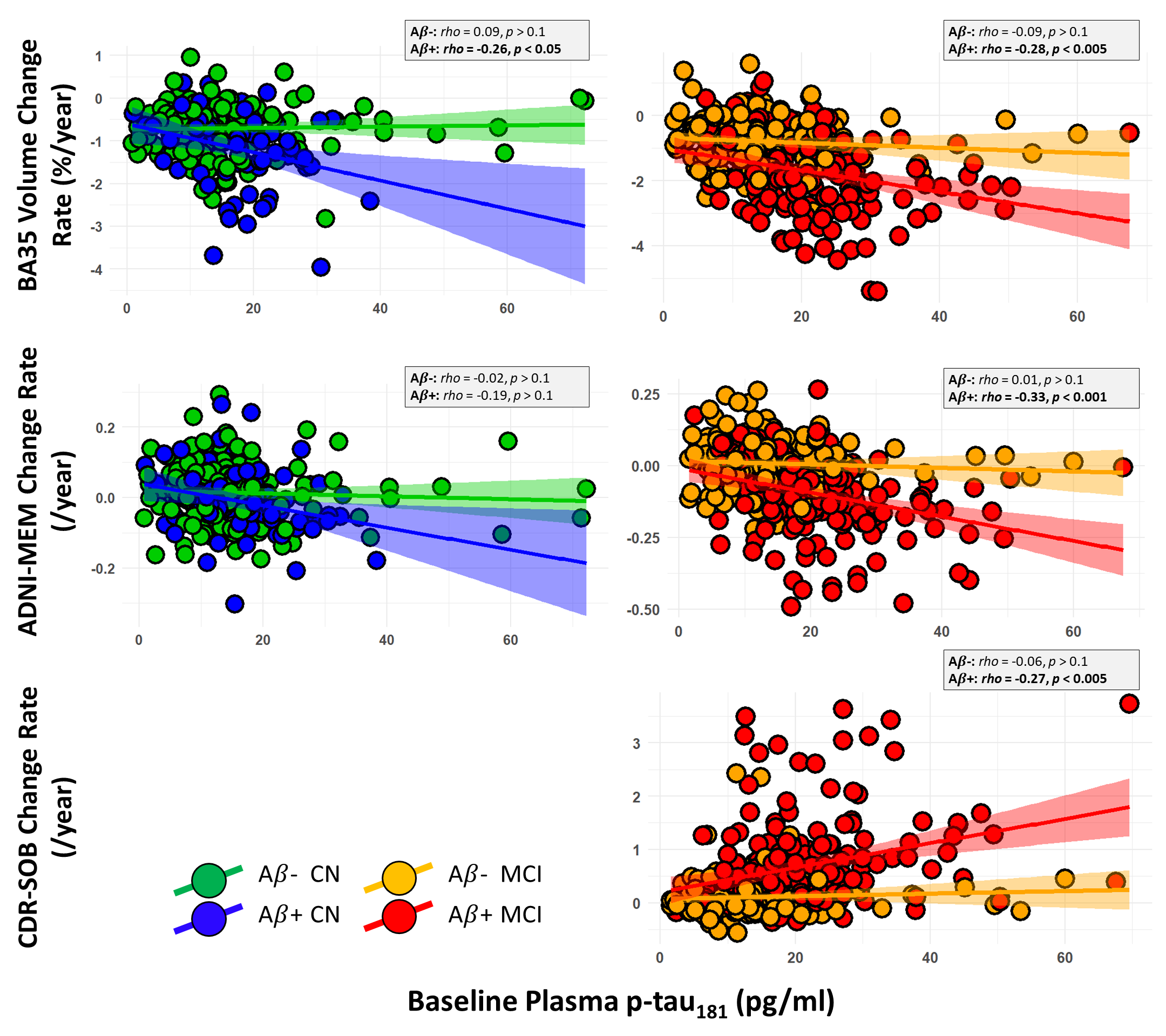


**Supplementary Figure S4.** Scatter plots of baseline plasma p-tau_181_ and all longitudinal measurements, corrected for age, sex, education, APOE ɛ4 status and intracranial volume. Abbreviations: CN = cognitive normal controls; MCI = mild cognitive impairment; CDR-SOB: clinical dementia rating sum-of-boxes; ADNI-MEM = ADNI summary memory score; BA35 = Brodmann area 35; p-tau = phosphorylated tau.

**Supplementary Table S2.** Results of the stepwise linear mixed effect modeling analyses for logical memory delayed recall (LDEL) in the All CN and All MCI groups (top), together with Aβ+ (middle) and Aβ- (bottom) subgroups. Variables that were fixed in the model: age, sex, education, intracranial volume and APOE ɛ4 status. Variables to be selected: baseline structural MRI measurements (highlighted in blue) and baseline plasma measurements (NfL and p-tau_181_, highlighted in orange).

| Dependent Variable | Group | Model statistics | Baseline measurements that are included in the model | |
| --- | --- | --- | --- | --- |
| All CN and MCI groups (regardless of Aβ status) | | | | |
| LDEL Change | All CN | N = 243  AIC = 4222.2  R^2^ = 0.63  AUC = 0.64 | PHC thickness | β = 0.17, p = 0.015 |
|  | All MCI | N = 352  AIC = 8024.19  R^2^ = 0.76  AUC = 0.88 | Plasma NfL  Posterior hippocampal volume  Plasma p-tau_181_  PHC thickness | β = -0.28, p = 2.9x10^-5^  β = 0.27, p = 4.0x10^-5^  β = -0.18, p = 6.8x10^-3^  β = 0.13, p = 0.043 |
| Aβ+ CN and MCI subgroups | | | | |
| LDEL Change | Aβ+ CN | N = 82  AIC = 1423.5  R^2^ = 0.73  AUC = 0.64 | None |  |
|  | Aβ+ MCI | N = 180  AIC = 4008.7  R^2^ = 0.79  AUC = 0.85 | Posterior hippocampal volume  Plasma p-tau_181_  Plasma NfL | β = 0.32, p = 2.9x10^-4^  β = -0.19, p = 0.027  β = -0.18, p = 0.045 |
| Aβ- CN and MCI subgroups | | | | |
| LDEL Change | Aβ- CN | N = 159  AIC = 2768.6  R^2^ = 0.55  AUC = 0.69 | PHC thickness | β = 0.23, p = 4.6x10^-3^ |
|  | Aβ- MCI | N = 164  AIC = 3777.0  R^2^ = 0.99  AUC = 0.85 | Plasma NfL  Posterior hippocampal volume  PHC thickness | β = -0.30, p = 4.3x10^-4^  β = 0.24, p = 8.6x10^-3^  β = 0.21, p = 0.020 |

Abbreviations: CN = cognitive normal controls; MCI = mild cognitive impairment; AUC = area under the curve; BA35 = Brodmann area 35; NfL = neurofilament light chain; p-tau = phosphorylated tau; PHC = parahippocampal cortex; AIC = Akaike information criterion.
